# Supplementary material for: The in vitro dynamics of pseudo-vascular network formation
Source: Br J Cancer. 2024 Jun 20;131(3):457–67. doi: 10.1038/s41416-024-02722-7 (PMC11300916; doi:10.1038/s41416-024-02722-7)
Supplement: Supplementary file 1 — Supplementary material [file 41416_2024_2722_MOESM1_ESM.pdf]

# Supplementary Information for:

# The *in vitro* dynamics of pseudo-vascular network formation

Mariam-Eleni Oraiopoulou [1,2], Dominique-Laurent Couturier [2,3], Ellie V. Bunce [1,2], Ian G. Cannell [2], Paul W. Sweeney [1,2], Huw Naylor [2], Monika Golinska [1,2], Gregory J. Hannon [2], Vangelis Sakkalis [4], Sarah E. Bohndiek [1,2,*]

**1** Department of Physics, University of Cambridge, Cambridge, UK

**2** Cancer Research UK Cambridge Institute (CRUK CI), University of Cambridge, Cambridge, UK

**3** Medical Research Council Biostatistics Unit, University of Cambridge, Cambridge, UK

**4** Institute of Computer Science, Foundation for Research and Technology – Hellas, Heraklion, Greece

***** Department of Physics, University of Cambridge, JJ Thomson Avenue, Cambridge, CB43 0HE and Cancer Research UK Cambridge Institute, University of Cambridge, Robinson Way, Cambridge, CB2 0RE, UK. seb53@cam.ac.uk. ORCID: 0000-0003-0371-8635.

**Keywords**

vasculogenic mimicry, tube formation assay, pseudo-vascular networks, morphometric analysis, descriptive statistical model

**Supplementary Figure S1**

Estimation of the effect of the endothelial cell growth basal medium-2 (EBM-2) and the basement membrane extract (BME) layer on growth or tubular formation.

(A) Exponential cell growth rate is impacted when the cells grow in the EBM-2, as compared to the respective cell culture medium for each of the cell lines, except for the B16-F10 cells, where both growth curves look similar. (B) The effect of the BME scaffold on the tubular formation was tested on the B16-F10 and the 4T1 cells, assuming that all the other cell lines will fall in between two extreme scenarios. For both the B16-F10 and the 4T1 cells, pseudo-vascular networks optimal formation for the respective timepoints can only be observed in 100% BME and endothelial culture medium presence; other combinations do not provide the right conditions for network formation.

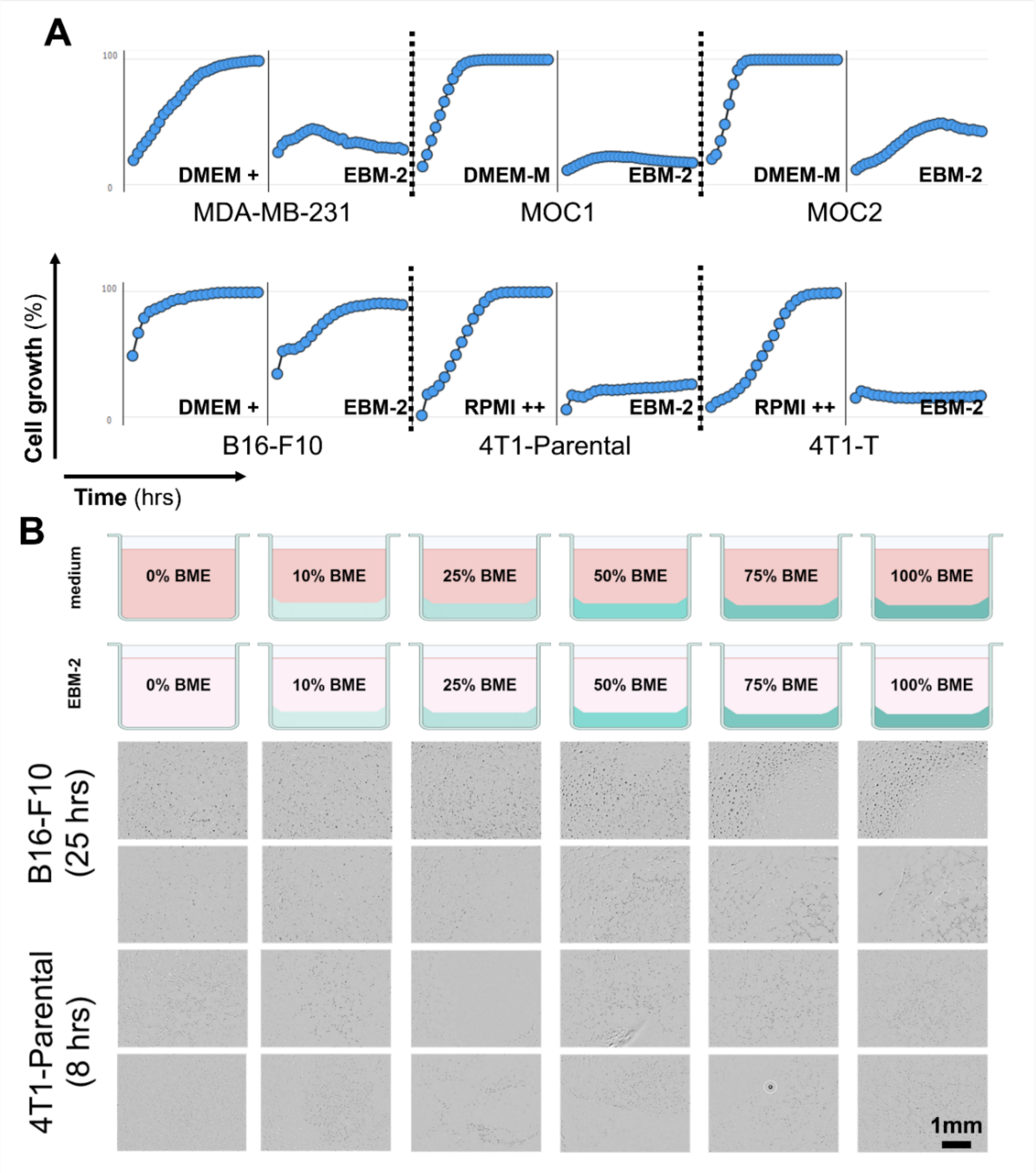

**Supplementary Figure S2**

Estimation of the average cell size for each of the cell lines tested in adherent and tube assay condition. (A) Exemplar phase contrast captures in 4x magnification of the two conditions for the MDA-MB-231 cell line (upper row) and the respective masks as estimated by Cellpose (low row). (B) Comparison of average cell size; dashed lines represent the mean values per cell culture condition. Data used for the analysis are from an intermediate timepoint of the respective time windows for pseudo-vascular network evolution and are therefore different for each cell line. (C) Confocal image of the 4T1 cells in adhesion (right) and in the pseudo-vascular network (left) as depicted at 10hrs in cell culture, respectively. Gross cell morphology and cell nuclei are stained with DAPI (blue), while cytoplasmic F-actin (magenta) and cell-to-cell adhesion E-cadherin (green) are also labelled.

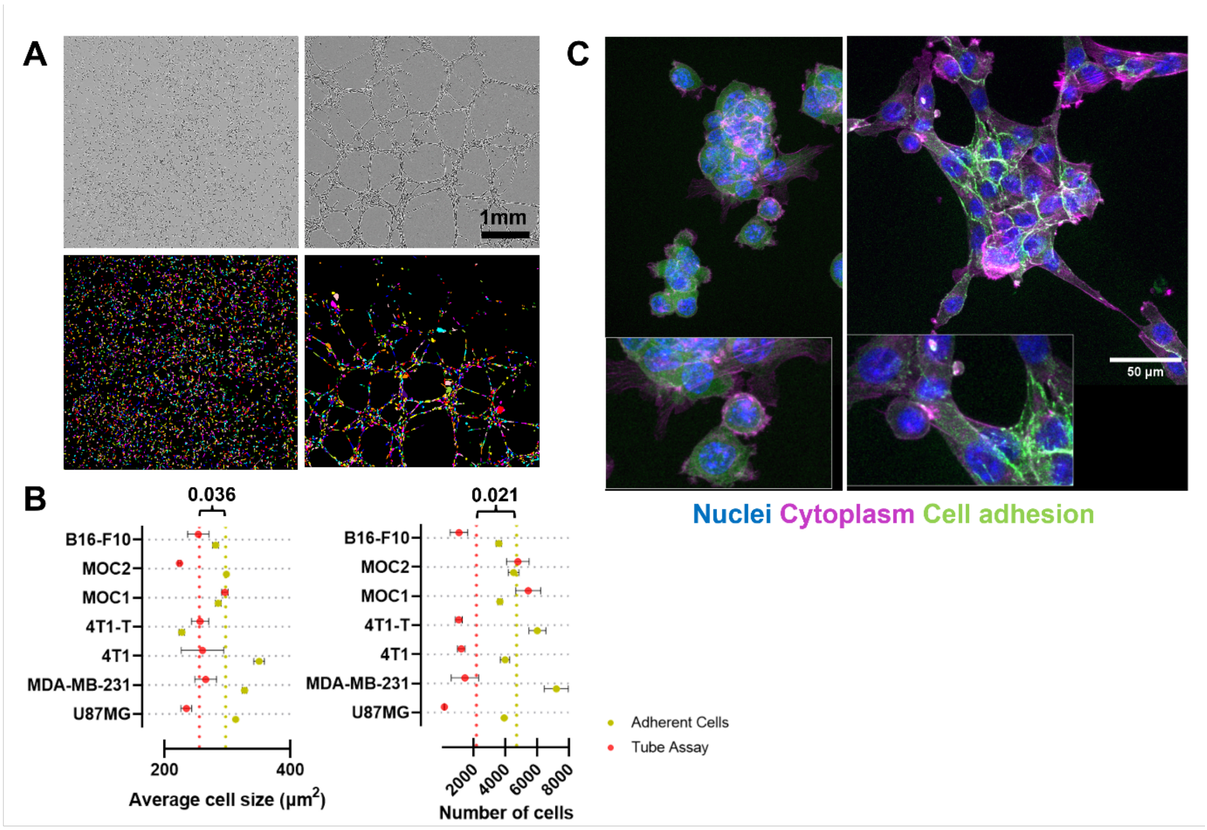

**Supplementary Figure S3**

Selected vectorial objects of the pseudo-vascular networks of the different cell lines over time, as described by the linear regression estimator.

1a - U87MG

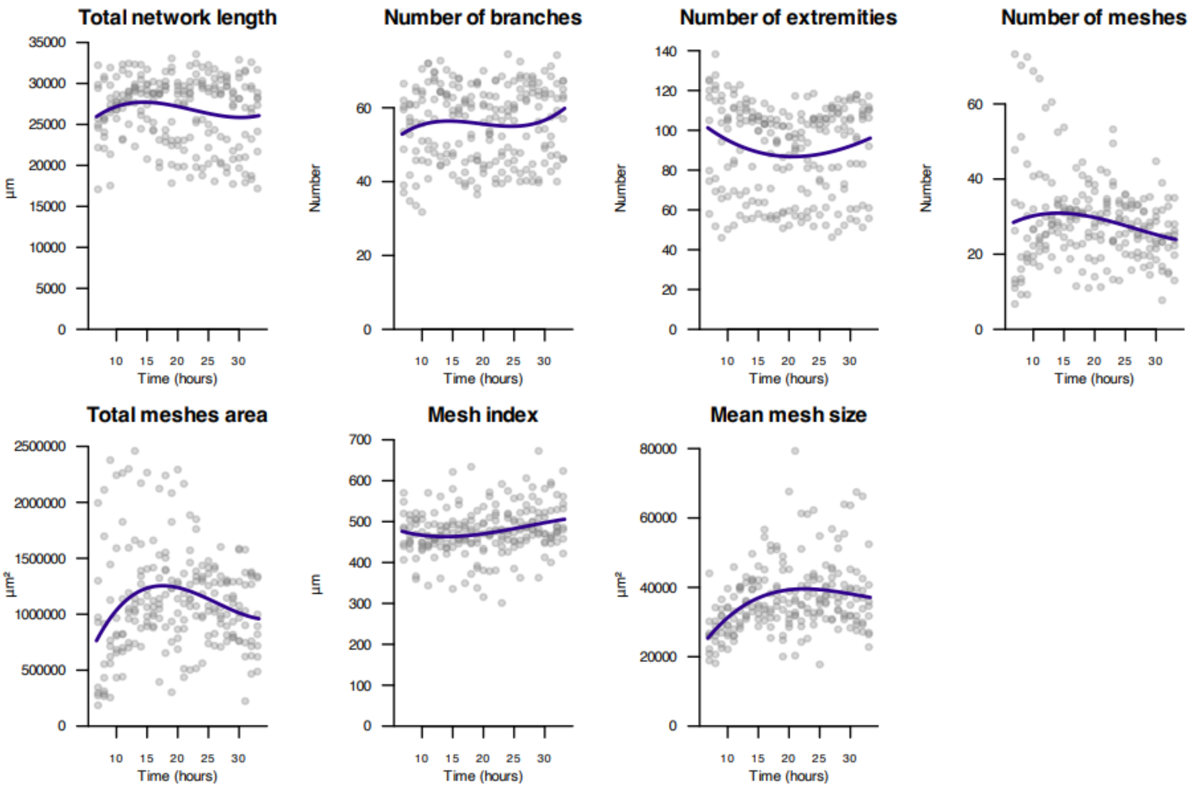

## 1b - MDA-MB-231

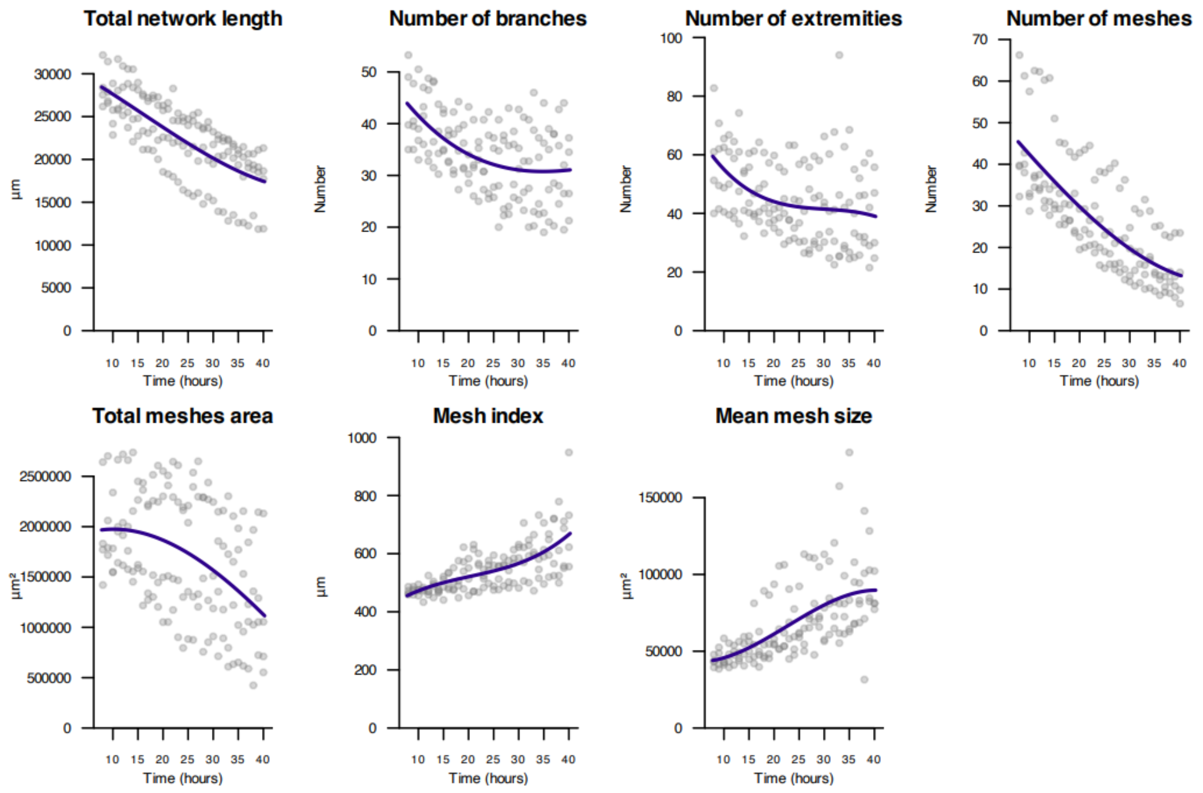


## 1c - 4T1

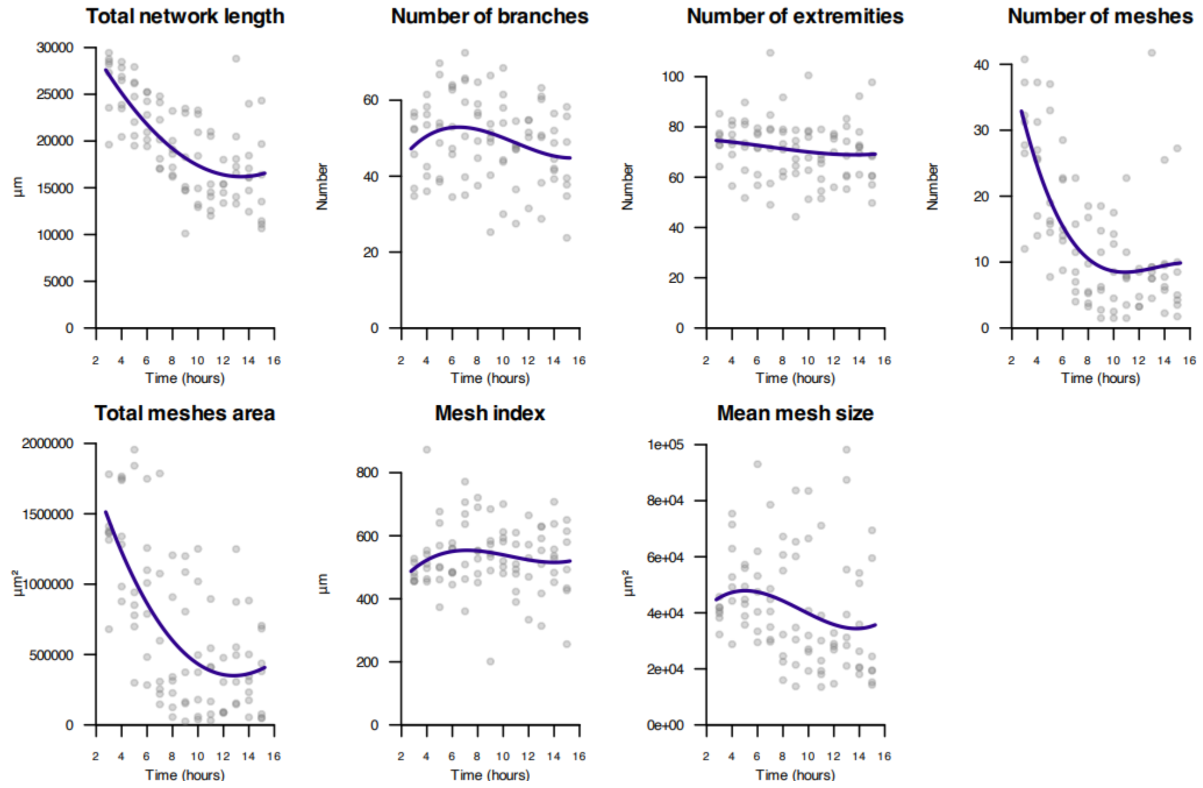

## 1d - 4T1-T

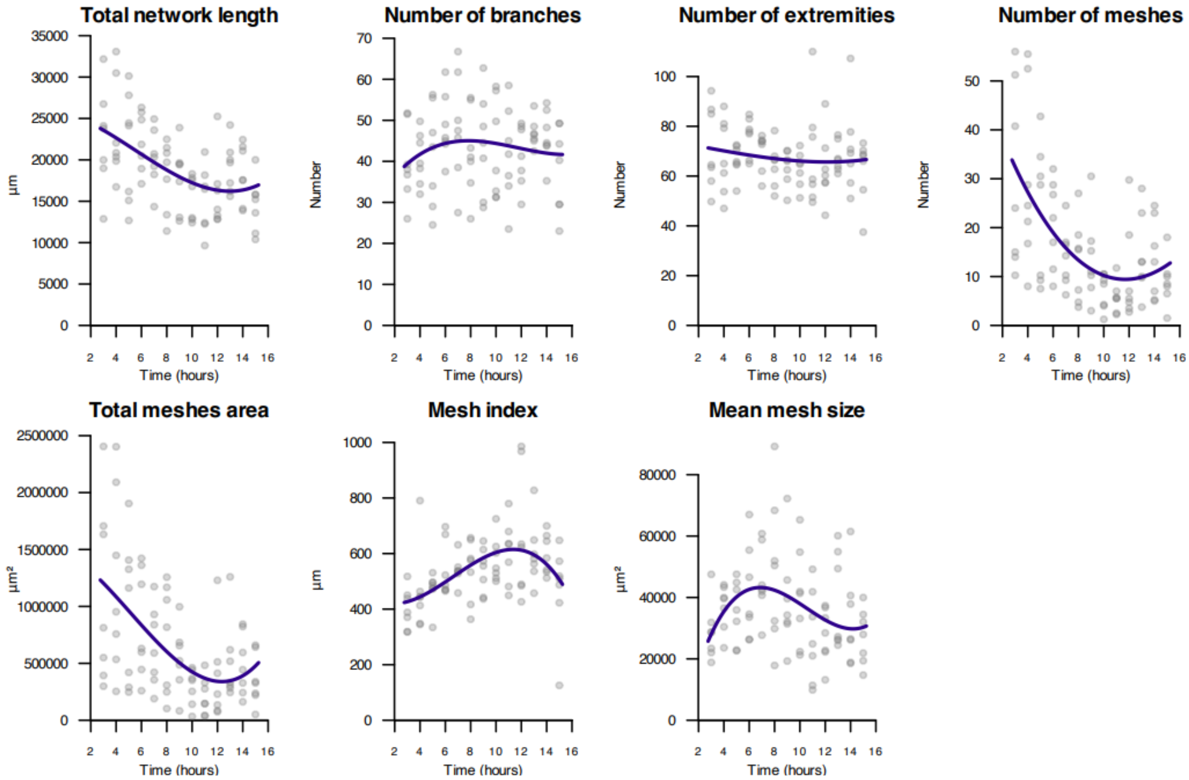

Total network length
µm
Time (hours)
Number of branches
Number
Time (hours)
Number of extremities
Number
Time (hours)
Number of meshes
Number
Time (hours)
Total meshes area
µm²
Time (hours)
Mesh index
µm
Time (hours)
Mean mesh size
µm²
Time (hours)

## 1e - B16-F10

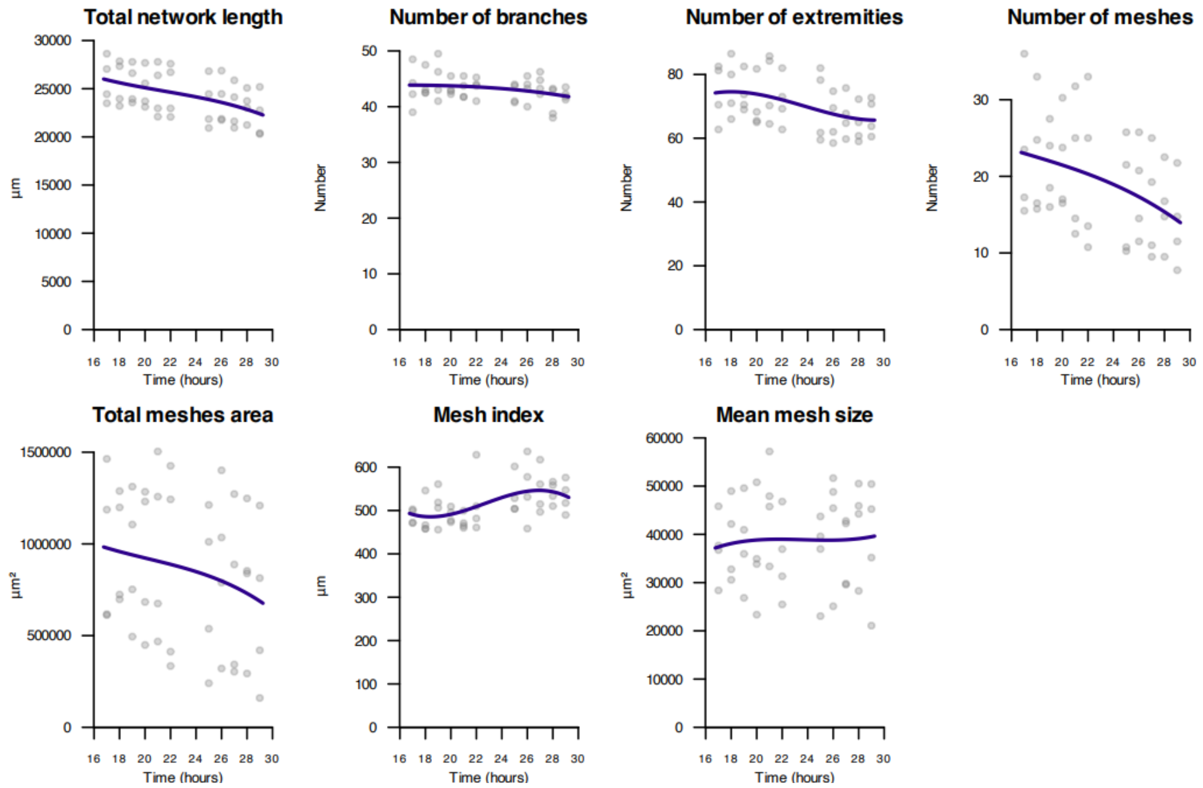
Total network length
µm
Time (hours)
Number of branches
Number
Time (hours)
Number of extremities
Number
Time (hours)
Number of meshes
Number
Time (hours)
Total meshes area
µm²
Time (hours)
Mesh index
µm
Time (hours)
Mean mesh size
µm²
Time (hours)

## 1f - MOC1

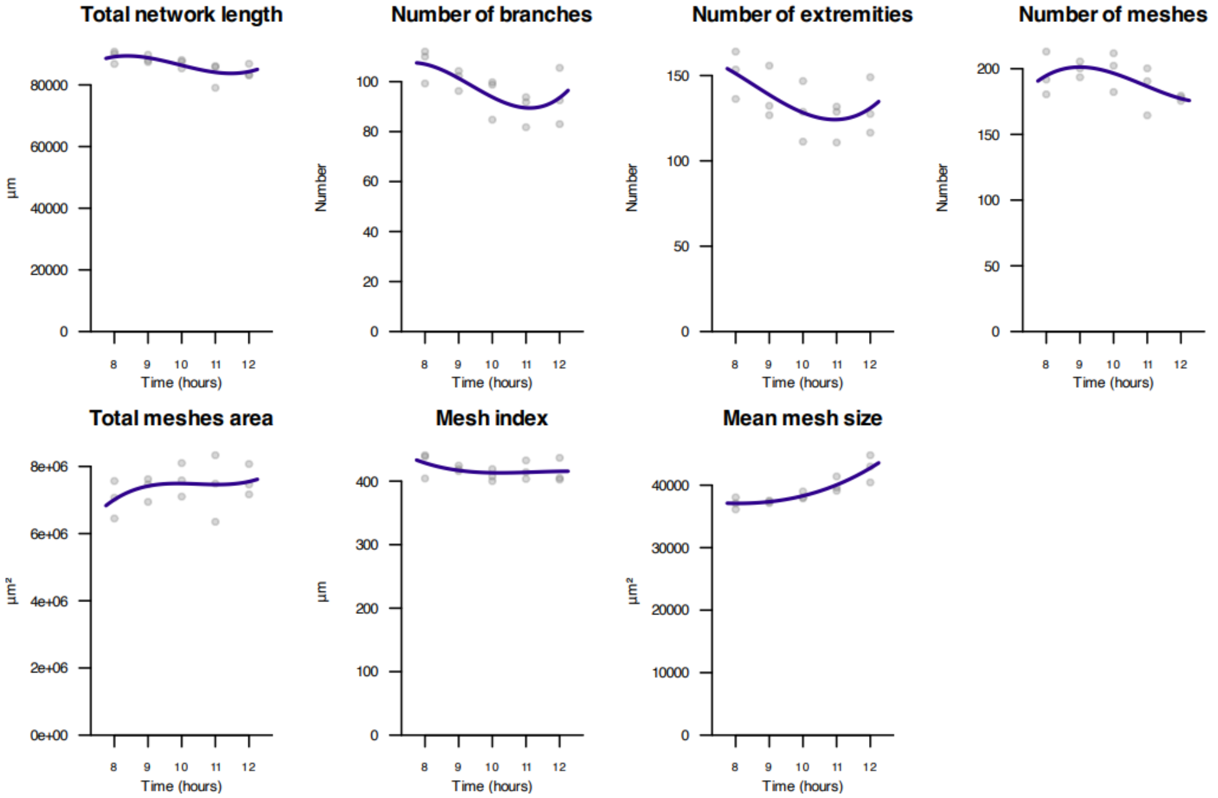


## 1g - MOC2

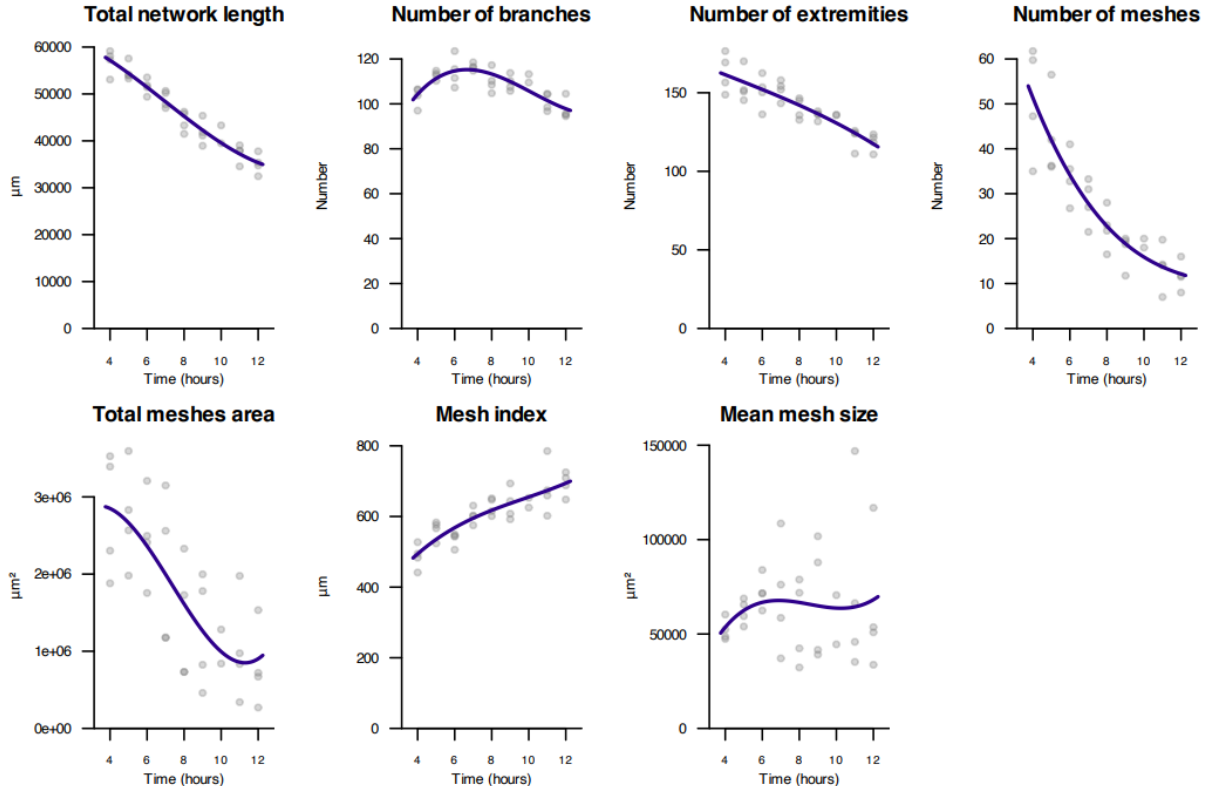

**Supplementary Figure S4**

Stability scores for each of the vectorial objects as described by the linear regression estimator (95% confidence interval width).

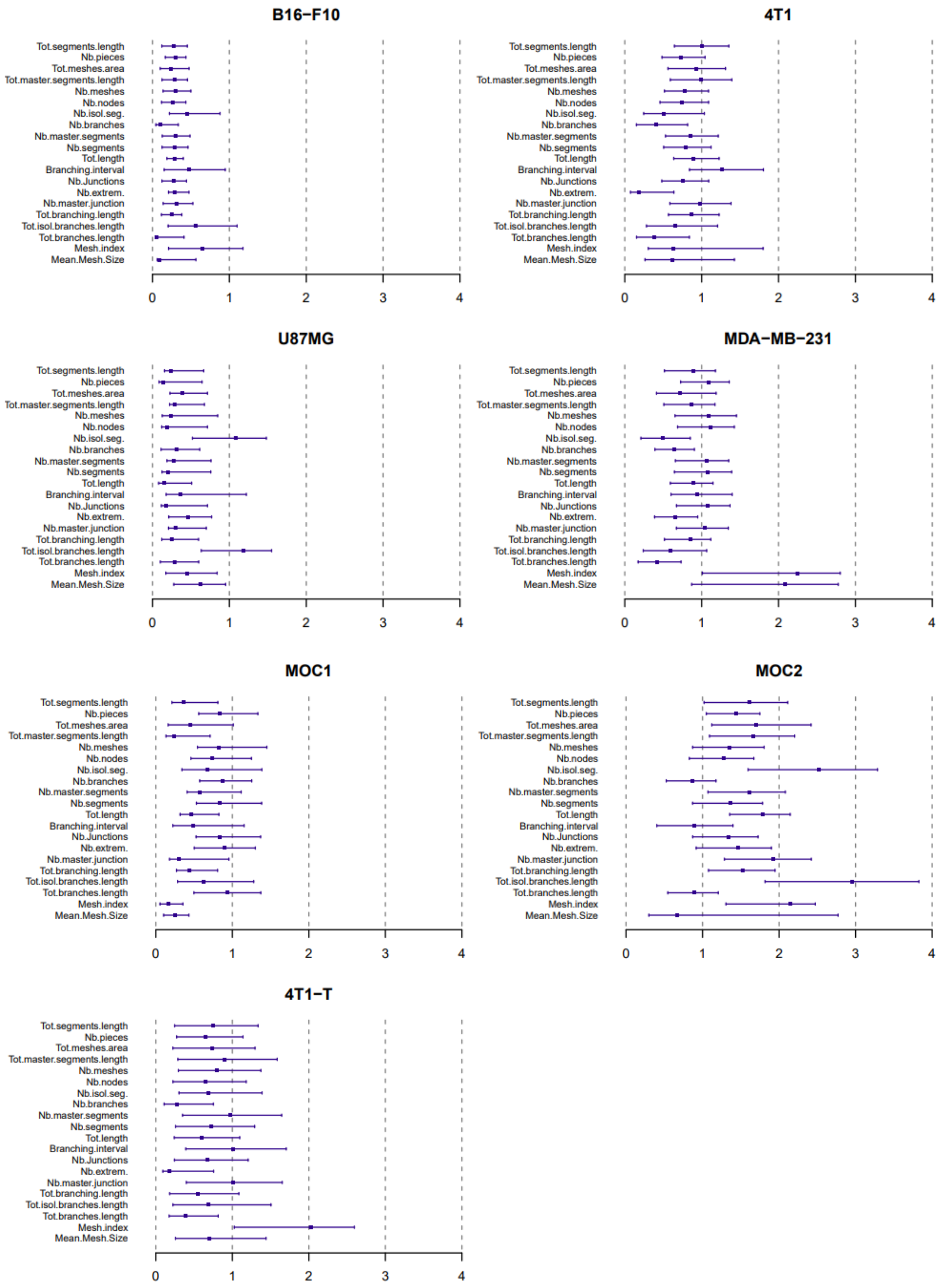

**Supplementary Figure S5**

The effect of the DMSO on the meshes of the pseudo-vascular networks of 4T1 and 4T1-T cell lines. DMSO concentrations other than 0.1% have been tested because of the effect of the DMSO on the tubular capacity. The 0.01% DMSO concentration seems to recapitulate the absence of any vehicle (control) more closely and therefore, it was used for the drug-treated tube formation assay.

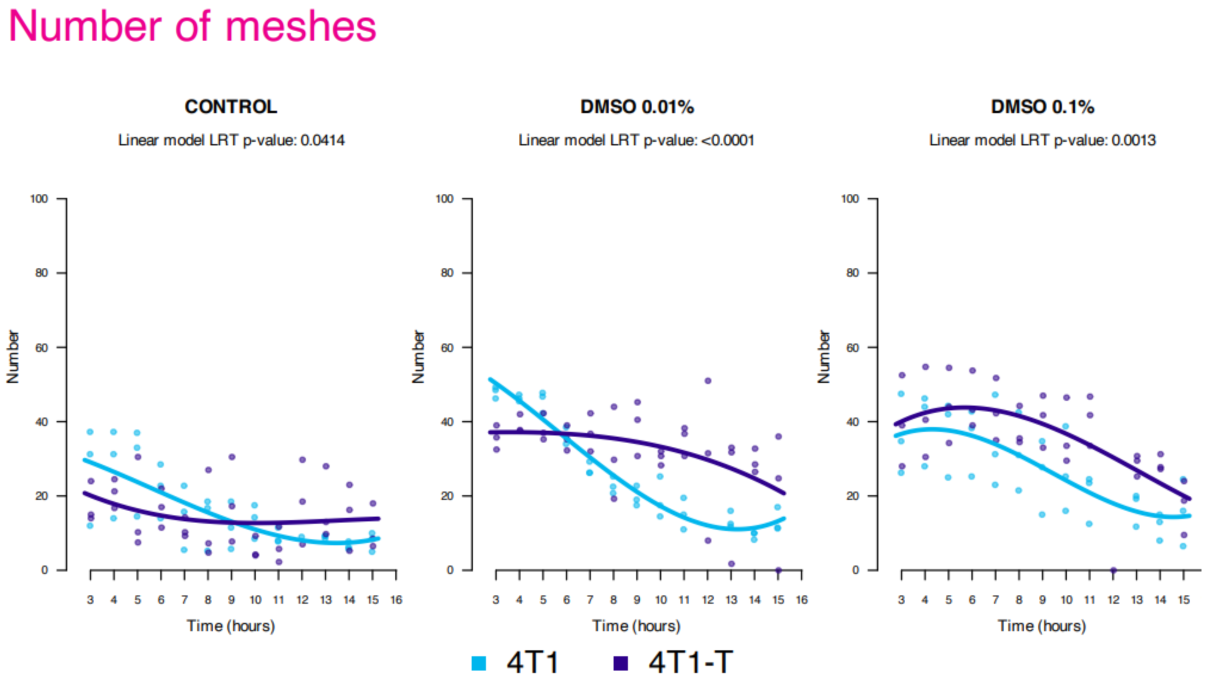

**Supplementary Figure S6**

To facilitate the comparison of the evolution of the cell lines for the different outcomes of interest, in the plots below an alternative representation of the plots of Figure 5 is provided considering standardised times on the x-axes.

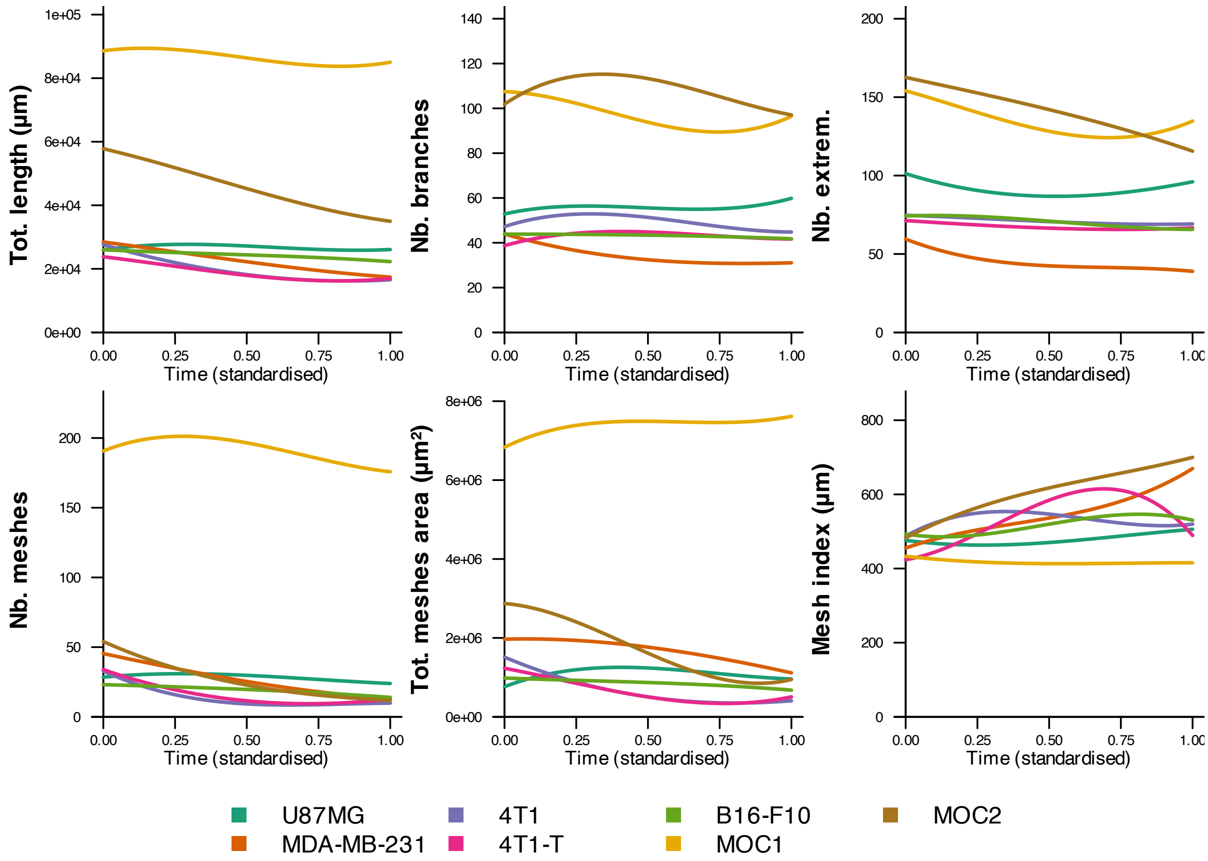

**Supplementary Table 1**

Definition of the pseudo-vascular network vectorial objects as defined in [19].

| Vectorial Object | Definition | Morphological Feature |
|---|---|---|
| **extremity** | ***pixel with only one neighbour*** | number of extremities |
| **node** | ***pixel with at least 3 neighbours*** | number of nodes |
| **junction** | ***group of dots forming a bifurcation*** | number of junctions<br>number of master junctions |
| **segment** | ***binary line linked with two junctions***<br><br>***(a piece is a segment intercepting a circle)*** | number of segments<br>number of master segments<br>total master segments length<br>number of isolated segments<br>total segments length<br>number of pieces |
| **branch** | ***line linked to one junction and one extremity*** | number of branches<br>total branching length<br>total branches length<br>total isolated branches length<br>branching interval |
| **mesh** | ***closed area formed by segments and their junctions***<br><br>***index: total master segments length / number of master segments*** | number of meshes<br>total mesh area<br>mean mesh size<br>mesh index |
| **network** | ***sum of length of segments, isolated elements and branches*** | total length |

**Supplementary Videos**

A sequence of phase-contrast images in 4x magnification, focusing on one quarter of a representative well for each of the cell lines, in order to monitor the cells undergoing the tube assay. Time slightly varies depending on the example, up to ~3 days post seeding. Acquisition is every 1hr and scale bar is set to 1mm. Videos have been made using the Incucyte software. Plate lid condensation, lost timepoints and changes in the focal plane can be expected during the automated process, as the experiments run uninterrupted for the whole time course.

Pseudo-vascular network time window is within this time period but is always concluded before 2 days for most of the cell lines and is often followed by a cell aggregation period. Changes in the confluence of the cells can be observed in the later phase of the time window, indicating proliferation of the cells after they are organised in tubes. Especially for the MOC2 cell line, there is evidence of proliferation (differentiation of the cellularity and the diameter of the tubes) during the organization into the pseudo-vascular network, but this is beyond the scope of this study.

An additional interactive video of the 3D reconstruction of the confocal image of the 4T1 pseudo-vascular network as shown in Supplementary Figure S2C is provided. Imaris software has been used to create this video.

**Video S1 –** U87MG

**Video S2 –** MDA-MB-231

**Video S3 –** 4T1

**Video S4 –** 4T1-T

**Video S5 –** MOC1

**Video S6 –** MOC2

**Video S7 –** B16-F10

**Video S8 –** 4T1 pseudo-vascular network confocal image
